# Carbohydrate-Dependent, Exercise-Induced Gastrointestinal Distress

**DOI:** 10.3390/nu6104191

**Published:** 2014-10-13

**Authors:** Erick Prado de Oliveira, Roberto C. Burini

**Affiliations:** 1 School of Medicine, Federal University of Uberlandia, Av. Pará, nº 1720 Bloco 2U, Campus Umuarama, Uberlandia, Minas Gerais 38400-902, Brazil; 2 Centre for Physical Exercise and Nutrition Metabolism, UNESP School of Medicine, Public Health Department, Botucatu City, São Paulo 18618-900, Brazil; E-Mail: burini@fmb.unesp.br

**Keywords:** carbohydrate, gastrointestinal problems, diet, exercise

## Abstract

Gastrointestinal (GI) problems are a common concern of athletes during intense exercise. Ultimately, these symptoms can impair performance and possibly prevent athletes from winning or even finishing a race. The main causes of GI problems during exercise are mechanical, ischemic and nutritional factors. Among the nutritional factors, a high intake of carbohydrate and hyperosmolar solutions increases GI problems. A number of nutritional manipulations have been proposed to minimize gastrointestinal symptoms, including the use of multiple transportable carbohydrates. This type of CHO intake increases the oxidation rates and can prevent the accumulation of carbohydrate in the intestine. Glucose (6%) or glucose plus fructose (8%–10%) beverages are recommended in order to increase CHO intake while avoiding the gastric emptying delay. Training the gut with high intake of CHO may increase absorption capacity and probably prevent GI distress. CHO mouth rinse may be a good strategy to enhance performance without using GI tract in exercises lasting less than an hour. Future strategies should be investigated comparing different CHO types, doses, and concentration in exercises with the same characteristics.

## 1. Introduction

The effect of exercise on the gastrointestinal (GI) tract depends mainly on the intensity and duration of exercise, while moderate intensity exercises have a protective effect [1], acute strenuous exercises cause gastrointestinal distress [2]. GI problems can impair performance and possibly prevent athletes from winning or even finishing a race [3].

There are several causes for gastrointestinal complaints during exercise, including mechanic, ischemic and nutritional factors [2,4]. During running and strength exercises, mechanic effects occur like enhanced intra-abdominal pressure and organs bouncing [5]. Additionally, during exercise, blood is shunted from viscera to active tissues (skeletal muscle, heart, lung and brain) [6,7] and splanchnic blood flow is decreased by as much as 80% and gastric emptying (GE) is thought to be negatively affected, especially when hypohydrated [7,8], which can cause gut mucosal ischemia and increases in mucosa permeability [5].

Nutrition can have a strong influence on gastrointestinal distress. Fiber, fat, protein, and concentrated carbohydrate (CHO) solutions have been associated with greater risk of GI distress [4,9]. Furthermore, depending of amount and type of CHO intake, it can also be a risk factor for GI distress.

During exercise, one of the contributors to fatigue is carbohydrate depletion [10]. Carbohydrate feeding can help to maintain plasma glucose concentration and prevent hypoglycemia; sparing hepatic glycogen, and in some cases delay muscle glycogen depletion [11]. This information can induce athletes to ingest large amounts of CHO and hyperosmolar beverage because a high intake of CHO is correlated with better performance. However, it can also lead to incomplete absorption and residual CHO increasing GI problems during exercise [12]. A number of nutritional manipulations have been proposed to minimize gastrointestinal symptoms, including use of multiple transportable carbohydrates [9]. This type of CHO intake increases oxidation rates and can prevent accumulation of carbohydrate in the intestine [13].

This review aimed to discuss the association of CHO intake and GI complaints during exercise, focusing on the type, amount and dilution of carbohydrate that can prevent these events.

## 2. CHO Intake and GI Problems

Carbohydrate intake, especially the excessive consumption of hypertonic drinks, is related to GI distress [14]. It has been proposed that hypertonic drinks cause GI distress via water retention to the human intestines [14] and, because of the excess of CHO, can lead to incomplete absorption. Moreover, residual CHO in the intestine has been linked to GI problems [15].

Individual CHO intake varies greatly among athletes (6–136 g/h). Ironman triathlon, half-Ironman, and marathon runners ingested 62 ± 26, 65 ± 25, and 35 ± 26 g/h of CHO intake during exercise and high intake of CHO is correlated with nausea (*r* = 0.34) and flatulence (*r* = 0.35) [12]. Pfeiffer *et al.* (2009) [16] reported higher prevalence of nausea after ingesting high (90 g/h) compared with lower amounts of CHO (60 g/h) during a 16-km outdoor run. In another study, flatulence was related to CHO consumption (sports drink beverage) when compared with water intake [17]. All these data suggest that CHO intake can increase the risk of nausea and flatulence during exercise [12].

Higher carbohydrate intake is correlated with flatulence and nausea, but it seems that there are not any negative effects on performance [12]. At this moment, no causal relationships can be obtained from these correlations because other factors, more than only carbohydrate intake, can be associated with GI distress, such as carbohydrate concentration, type of carbohydrate, osmolality and acidity [9]. Furthermore, different methodologies and statistical approaches were used in each study [16]. Future studies are needed to investigate the main GI problems associated with CHO intake.

The form of carbohydrate intake is generally solid, gel and liquid. CHO gels and CHO-containing drinks are main source of CHO during races, whereas solid form is the least ingested [12]. All CHO forms can be ingested during exercise because there is no difference in exogenous CHO oxidation when compared to intake of solid, gel and, drink forms; however, consumption of solid CHO increases stomach fullness sensation when compared to gel and drink forms [18,19] and should be avoided mainly in individuals with a history of GI problems.

## 3. CHO Beverage Concentration

During competition, optimal CHO beverage concentration seems to be in the range of 5%–8%, and athletes should aim to achieve a CHO intake of 60–70 g/h. Lower gastric emptying is directly associated with GI problems [20] and effects of increasing carbohydrate concentration content upon fluid delivery should be studied. Healthy male seated subjects ingested 0%, 3%, 6%, and 9% CHO beverage content upon fluid delivery. It was concluded that fluid delivery was compromised when carbohydrate beverage was increased above 6%. Furthermore, it was possible to note that 3% CHO beverage has faster fluid delivery than water [21].

The effectiveness of different carbohydrate solutions in restoring fluid balance in situations of voluntary fluid intake was examined in 1.99% body mass dehydrated (intermittent route) subjects [22]. Beginning 30 min after cessation of exercise, subjects drank *ad libitum* for a period of 120 min, drinks containing 31 mmol/L sodium as NaCl and either 0%, or 2% or 10% glucose. Lower concentration (0% and 2%) resulted in larger plasma volume than 10% glucose intake and authors concluded hypertonic carbohydrate-electrolyte solutions are as effective as hypotonic carbohydrate-electrolyte solutions at restoring whole-body fluid balance and no difference was observed in GI complaints among the trials [22]. It could be recommended mainly after exercise because there is no more blood flow redistribution from viscera to muscle. If intake occurred during exercise, higher GI complaints could probably be observed with hypertonic beverages.

In another study, 36 adult and adolescent athletes were tested on separate days in a double-blind, randomized trial of 6% and 8% carbohydrate-electrolytes beverages during four 12-min quarters of circuit training including intermittent sprints, lateral hops, shuttle runs and vertical jumps. GI discomfort were tested and it was concluded that 8% CHO showed higher ratings of stomach upset and side ache [23]. The weakness of this study was that the type of CHO that was different between two beverages and the correct aim would be to compare only CHO concentration, with the same types of CHO (e.g., only glucose).

## 4. Types of CHO

### 4.1. CHO of Multiple Transporters

Multiple transporters CHO intake (glucose + fructose) increases absorption and fluid delivery because glucose and fructose are absorbed by different transporters. Glucose transport across the brush border occurs by sodium-dependent glucose transporter (SGLT1), whereas fructose is absorbed by GLUT5 [24].

To avoid GI complaints, it is more logical to recommend consumption of multiple transporters of CHO, because, when comparing a 8.6% glucose solution (glucose) with 8.6% (glucose + fructose) solution (90 g/h) it is observed that glucose plus fructose increases GE and fluid delivery [25]. Even after 5 h of exercise, a high intake of glucose plus fructose (90 g/h) does not increase stomach fullness sensation, which is observed with glucose intake [26].

A CHO intake in the form of multiple transportable carbohydrates (glucose plus fructose) can be ingested at rate of 1.5 g/min [25] to 1.8 g/min [27]. A solution intake with 1.2 g/min of maltodextrin + 0.6 g/min of fructose shows higher carbohydrate oxidation (approximately 1.5 g/min) than 1.8 g/min of maltodextrin (alone) [27].

There is still no consensus of ideal glucose: fructose ratio intake to prevent GI problems. However, a recent study tested a high intake of CHO (1.8 g/min) in 10 cyclists that rode 150 min at 50% peak power until exhaustion and compared proportion of fructose and maltodextrin: 0.6 fructose + 1.2 maltodextrin (0.5 ratio), 0.8 fructose + 1.0 maltodextrin (0.8 ratio) and 1.0 fructose + 0.8 maltodextrin (1.25 ratio) on GI complaints and performance. Authors concluded that 0.8 ratio showed lower ratings of gastrointestinal discomfort (stomach fullness and nausea) and this was also the main factor to enhance the performance [28]. More studies are necessary comparing glucose: fructose ratio intake in different exercises, intensities and modalities.

### 4.2. Single vs. Multiple CHO Transporters

**Figure 1:**
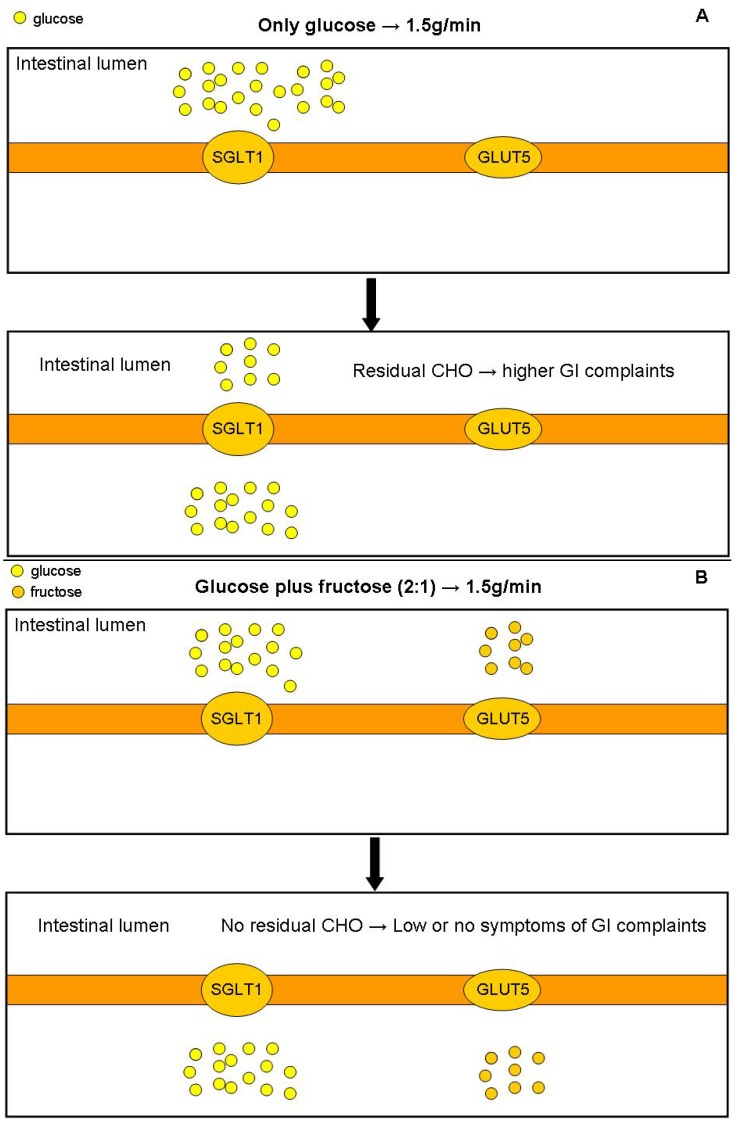
Comparison of absorption between high intake of glucose (**A**) and glucose plus fructose (**B**).

Multiple CHO transporters increase fluid delivery and higher amounts can be ingested during exercise. It could be an optimal strategy to decrease residual CHO in the gastrointestinal tract during exercise and consequently to avoid GI complaints (Figure 1). Ingestion of multiple transportable *vs.* single transportable carbohydrates enhances exogenous CHO oxidation and therefore increases performance [29], which can also be explained by reduction in gastrointestinal discomfort [30]. To increase CHO intake avoiding gastric emptying delay and intestinal residual CHO, the recommendation could be glucose (6% beverage or 1.0 g/min) or glucose plus fructose (8%–10% beverage or 1.5–1.8 g/min). The main limitations of the studies using multiple CHO transporters beverage to decrease GI problems are different kinds of exercises, intensities and duration. Because of this, more research needs to be conducted.

## 5. Carbohydrate Mouth Rinse

**Figure 2:**
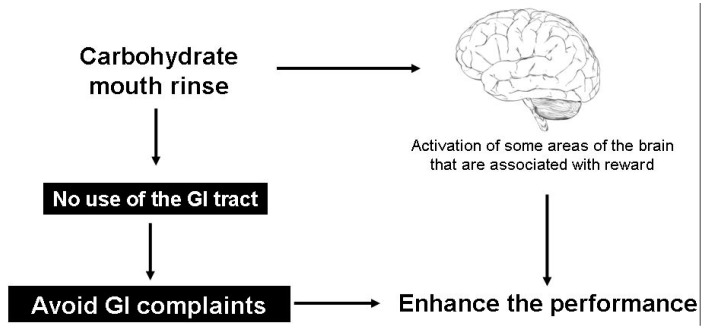
Effect of carbohydrate mouth rinse in gastrointestinal tract and performance.

A strategy to prevent GI distress during exercise could be to not use the GI tract to avoid gastric emptying delay caused mainly by concentrated CHO beverages. It is known that CHO intake enhances performance [29], but there is evidence that it is not necessary to intake large amounts of CHO during exercise lasting less than 60 min. Mouth rinse with CHO can be a good choice to enhance performance [31] without using the GI tract (Figure 2). These effects occur because there are some receptors in the oral cavity that activate some cerebral areas associated with reward [32].

Most studies used a maltodextrin or glucose beverage and a 1-h running test, cycling time trial, or time to exhaustion to evaluate the performance [11]. Duration of mouth rinse has already been discussed. In a recent study, Sinclair *et al.* [33] compared the effect of different durations of CHO mouth rinse on cycling performance, comparing 5 and 10 seconds durations with 6.4% CHO affected 30-min self-selected cycling performance. An improvement in distance cycled within 10 s was observed. There appears to be an ideal duration of mouth rinse to result in greater period of brain areas activations linked to motivation and motor control.

Studies have shown improvements between 2% and 3% during exercise lasting approximately 1 h. Effects are observed to be more profound after an overnight fast and, when fed, the effects of a mouth rinse are diminished [11]. This could be an important practice to enhance performance, because this improvement is significant in elite athletes. Further research is necessary to test the advantage of CHO mouth rinse to decrease GI problems during exercises.

## 6. Trainability of Gut with High Intake of Carbohydrate

Since higher absorption of CHO is associated with less residual CHO in the intestine and it could prevent GI distress, an obvious potential strategy would be to increase absorptive capacity of the gut.

A recent study proposed that the gut is trainable and individuals who regularly consume carbohydrate or have a high daily intake of CHO may increase absorption capacity [34]. Sixteen endurance-trained cyclists or triathletes were pair matched and randomly allocated to either a high-carbohydrate group (*n* = 8) or an energy-matched low-carbohydrate group (*n* = 8) for 28 days. Authors concluded that the high-carbohydrate group experienced enhanced total CHO oxidation after a month of “gut training”, suggesting that the intestine is trainable and can be adapted to absorb more CHO.

Considering that exogenous CHO is limited mainly by the rate of absorption and subsequent transport of ingested CHO to circulation than rate of muscle uptake [35], and that no difference in muscle GLUT-4 concentration was found between the groups [34], a possible mechanism is that high CHO intake can increase intestinal absorption capacity. An animal study has shown that a high-glucose diet stimulated glucose transport activity and increased levels of SGLT1 [36]. However, further work is needed in humans to confirm this hypothesis. Furthermore, more studies are also needed to clarify if “gut training” can decrease GI distress symptoms, mainly in individuals with a history of GI complaints.

## 7. Conclusions

Gastrointestinal problems during exercise are common among athletes and the correct concentration of CHO beverage and type of CHO are important factors to avoid GI problems. Ingestion of multiple transportable carbohydrates seems to be a good strategy to improve performance, probably because of reduction in GI distress because of a lower amount of residual carbohydrate in the intestine. Some nutritional interventions such as gut training and CHO mouth rinse should be studied to find out if such interventions could reduce GI problems during the exercise.

## References

[1] Dainese R., Serra J., Azpiroz F., Malagelada J.R. (2004). Effects of physical activity on intestinal gas transit and evacuation in healthy subjects. Am. J. Med..

[2] De Oliveira E.P., Burini R.C. (2009). The impact of physical exercise on the gastrointestinal tract. Curr. Opin. Clin. Nutr. Metab. Care.

[3] Peters H.P., Bos M., Seebregts L., Akkermans L.M., van Berge Henegouwen G.P., Bol E., Mosterd W.L., de Vries W.R. (1999). Gastrointestinal symptoms in long-distance runners, cyclists, and triathletes: prevalence, medication, and etiology. Am. J. Gastroenterol..

[4] De Oliveira E.P., Burini R.C. (2011). Food-dependent, exercise-induced gastrointestinal distress. J. Int. Soc. Sports Nutr..

[5] Casey E., Mistry D.J., MacKnight J.M. (2005). Training room management of medical conditions: Sports gastroenterology. Clin. Sports Med..

[6] Ogoh S. (2008). Autonomic control of cerebral circulation: Exercise. Med. Sci. Sports Exerc..

[7] Qamar M.I., Read A.E. (1987). Effects of exercise on mesenteric blood flow in man. Gut.

[8] Leiper J.B., Broad N.P., Maughan R.J. (2001). Effect of intermittent high-intensity exercise on gastric emptying in man. Med. Sci. Sports Exerc..

[9] De Oliveira E.P., Burini R.C., Jeukendrup A. (2014). Gastrointestinal complaints during exercise: Prevalence, etiology, and nutritional recommendations. Sports Med..

[10] Jeukendrup A.E. (2011). Nutrition for endurance sports: marathon, triathlon, and road cycling. J. Sports Sci..

[11] Jeukendrup A.E. (2013). Oral carbohydrate rinse: placebo or beneficial?. Curr. Sports Med. Rep..

[12] Pfeiffer B., Stellingwerff T., Hodgson A.B., Randell R., Pottgen K., Res P., Jeukendrup A.E. (2012). Nutritional intake and gastrointestinal problems during competitive endurance events. Med. Sci. Sports Exerc..

[13] Jeukendrup A. (2014). A step towards personalized sports nutrition: carbohydrate intake during exercise. Sports Med..

[14] Rehrer N.J., van Kemenade M., Meester W., Brouns F., Saris W.H. (1992). Gastrointestinal complaints in relation to dietary intake in triathletes. Int. J. Sport Nutr..

[15] Rehrer N.J., Wagenmakers A.J., Beckers E.J., Halliday D., Leiper J.B., Brouns F., Maughan R.J., Westerterp K., Saris W.H. (1992). Gastric emptying, absorption, and carbohydrate oxidation during prolonged exercise. J. Appl. Physiol. (1985).

[16] Pfeiffer B., Cotterill A., Grathwohl D., Stellingwerff T., Jeukendrup A.E. (2009). The effect of carbohydrate gels on gastrointestinal tolerance during a 16-km run. Int. J. Sport Nutr. Exerc. Metab..

[17] Van Nieuwenhoven M.A., Brouns F., Kovacs E.M. (2005). The effect of two sports drinks and water on GI complaints and performance during an 18-km run. Int. J. Sports Med..

[18] Pfeiffer B., Stellingwerff T., Zaltas E., Jeukendrup A.E. (2010). CHO oxidation from a CHO gel compared with a drink during exercise. Med. Sci. Sports Exerc..

[19] Pfeiffer B., Stellingwerff T., Zaltas E., Jeukendrup A.E. (2010). Oxidation of solid *versus* liquid CHO sources during exercise. Med. Sci. Sports Exerc..

[20] Brouns F., Beckers E. (1993). Is the gut an athletic organ? Digestion, absorption and exercise. Sports Med..

[21] Jeukendrup A.E., Currell K., Clarke J., Cole J., Blannin A.K. (2009). Effect of beverage glucose and sodium content on fluid delivery. Nutr. Metab. (Lond.).

[22] Evans G.H., Shirreffs S.M., Maughan R.J. (2009). Postexercise rehydration in man: The effects of carbohydrate content and osmolality of drinks ingested ad libitum. Appl. Physiol. Nutr. Metab..

[23] Shi X., Horn M.K., Osterberg K.L., Stofan J.R., Zachwieja J.J., Horswill C.A., Passe D.H., Murray R. (2004). Gastrointestinal discomfort during intermittent high-intensity exercise: Effect of carbohydrate-electrolyte beverage. Int. J. Sport Nutr. Exerc. Metab..

[24] Wright E.M., Martin M.G., Turk E. (2003). Intestinal absorption in health and disease—Sugars. Best Pract. Res. Clin. Gastroenterol..

[25] Jeukendrup A.E., Moseley L. (2010). Multiple transportable carbohydrates enhance gastric emptying and fluid delivery. Scand. J. Med. Sci. Sports.

[26] Jeukendrup A.E., Moseley L., Mainwaring G.I., Samuels S., Perry S., Mann C.H. (2006). Exogenous carbohydrate oxidation during ultraendurance exercise. J. Appl. Physiol..

[27] Wallis G.A., Rowlands D.S., Shaw C., Jentjens R.L., Jeukendrup A.E. (2005). Oxidation of combined ingestion of maltodextrins and fructose during exercise. Med. Sci. Sports Exerc..

[28] O’Brien W.J., Rowlands D.S. (2011). Fructose-maltodextrin ratio in a carbohydrate-electrolyte solution differentially affects exogenous carbohydrate oxidation rate, gut comfort, and performance. Am. J. Physiol. Gastrointest. Liver Physiol..

[29] Jeukendrup A.E. (2010). Carbohydrate and exercise performance: The role of multiple transportable carbohydrates. Curr. Opin. Clin. Nutr. Metab. Care.

[30] Rowlands D.S., Swift M., Ros M., Green J.G. (2012). Composite *versus* single transportable carbohydrate solution enhances race and laboratory cycling performance. Appl. Physiol. Nutr. Metab..

[31] Jeukendrup A.E., Chambers E.S. (2010). Oral carbohydrate sensing and exercise performance. Curr. Opin. Clin. Nutr. Metab. Care.

[32] Chambers E.S., Bridge M.W., Jones D.A. (2009). Carbohydrate sensing in the human mouth: Effects on exercise performance and brain activity. J. Physiol..

[33] Sinclair J., Bottoms L., Flynn C., Bradley E., Alexander G., McCullagh S., Finn T., Hurst H.T. (2014). The effect of different durations of carbohydrate mouth rinse on cycling performance. Eur. J. Sport Sci..

[34] Cox G.R., Clark S.A., Cox A.J., Halson S.L., Hargreaves M., Hawley J.A., Jeacocke N., Snow R.J., Yeo W.K., Burke L.M. (2010). Daily training with high carbohydrate availability increases exogenous carbohydrate oxidation during endurance cycling. J. Appl. Physiol..

[35] Jeukendrup A.E. (2004). Carbohydrate intake during exercise and performance. Nutrition.

[36] Miyamoto K., Hase K., Takagi T., Fujii T., Taketani Y., Minami H., Oka T., Nakabou Y. (1993). Differential responses of intestinal glucose transporter mRNA transcripts to levels of dietary sugars. Biochem. J..

